# Immunization of Mice with Lentiviral Vectors Targeted to MHC Class II+ Cells Is Due to Preferential Transduction of Dendritic Cells *In Vivo*

**DOI:** 10.1371/journal.pone.0101644

**Published:** 2014-07-24

**Authors:** Séverine Ciré, Sylvie Da Rocha, Roseline Yao, Sylvain Fisson, Christian J. Buchholz, Mary K. Collins, Anne Galy

**Affiliations:** 1 Inserm, U 951, Molecular Immunology and Innovative Biotherapies, Genethon, Evry, France; 2 UMR_S951, University of Evry, Genethon, Evry, France; 3 Genethon, Evry, France; 4 Molecular Biotechnology and Gene Therapy, Paul-Ehrlich-Institut, Langen, Germany; 5 Infection and Immunity, University College London, London, United Kingdom; University of Pittsburgh School of Medicine, United States of America

## Abstract

Gene transfer vectors such as lentiviral vectors offer versatile possibilities to express transgenic antigens for vaccination purposes. However, viral vaccines leading to broad transduction and transgene expression *in vivo,* are undesirable. Therefore, strategies capable of directing gene transfer only to professional antigen-presenting cells would increase the specific activity and safety of genetic vaccines. A lentiviral vector pseudotype specific for murine major histocompatibilty complex class II (LV-MHCII) was recently developed and the present study aims to characterize the *in vivo* biodistribution profile and immunization potential of this vector in mice. Whereas the systemic administration of a vector pseudotyped with a ubiquitously-interacting envelope led to prominent detection of vector copies in the liver of animals, the injection of an equivalent amount of LV-MHCII resulted in a more specific biodistribution of vector and transgene. Copies of LV-MHCII were found only in secondary lymphoid organs, essentially in CD11c+ dendritic cells expressing the transgene whereas B cells were not efficiently targeted *in vivo,* contrary to expectations based on *in vitro* testing. Upon a single injection of LV-MHCII, naive mice mounted specific effector CD4 and CD8 T cell responses against the intracelllular transgene product with the generation of Th1 cytokines, development of *in vivo* cytotoxic activity and establishment of T cell immune memory. The targeting of dendritic cells by recombinant viral vaccines must therefore be assessed *in vivo* but this strategy is feasible, effective for immunization and cross-presentation and constitutes a potentially safe alternative to limit off-target gene expression in gene-based vaccination strategies with integrative vectors.

## Introduction

Gene-specific immunization is a promising concept in vaccination owing to the versatility of genetic constructs that can be engineered to express immunogens in various and complex forms. Recombinant viral vector systems, such as lentiviral vectors (LV), have already been used effectively as genetic vaccines notably to express and immunize against non-secreted cellular antigens in cancer or infectious disease applications [1], [2]. Effective T cell immunization is initiated by antigenic presentation to naïve T cells by professional antigen-presenting cells (APCs) such as dendritic cells (DCs). Thus, directing gene delivery distinctively to APC, moreover to DC, is an attractive concept to augment the specific activity of genetic vaccines and to reduce the risks of adverse reactions such as auto-immunity or immune tolerance that could result from persistent antigenic expression in an inadequate compartment [3]. Targeting genetic vaccines to a relatively non-abundant population of specialized cells such as DC would also in effect reduce the risk of genotoxicity inherent to approaches based on integrative vector.

Enveloped viral vectors such as LV provide possibilities for cell-targeting though the use of engineered envelope glycoproteins exploiting either the natural tropisms of viral glycoproteins [4] or by engineering artificial targeting constructs [5]. Recently, a ligand-specific pseudotyping platform was derived from modified measles virus (MV) glycoproteins by mutating its natural ligands - CD46 and SLAM - recognition sites and inserting a single chain immunoglobulin variable region fragment (ScFv) in the C-terminal region of the H chain to retarget the particles to specific moieties [6]. The identification of a ScFv specific for a non polymorphic determinant on the α chain of the mouse MHC-II was exploited in this platform to generate LVs targeting MHC class II+ cells (LV-MHCII) [7], [8]. In tissue culture, LV-MHCII specifically transduce MHC class II+ cells which include CD11c+ DC, CD19+ B cells and F4/80 CD11b+ macrophages. When injected to mice, LV-MHCII encoding ovalbumin generated a specific immune response with IFN-γ production in spleen cells [7]. However, further characterization of the system is required to determine if a fully effective T cell response can be achieved with this vector and to analyze its activity in relation to the vector *in vivo* biodistribution pattern and targeting of various populations of MHCII+ cells in lymphoid organs.

To address these questions, we developed a novel antigenic system enabling the detection of transduced APCs and of transgen-specific T cell immune responses from the same construct. The antigen is a fusion of the enhanced green fluorescent protein (GFP) with CD4 and CD8 T cell epitopes of the murine male gene HY (GFP-HY) which are immunogenic in female mice. Using vectors produced by standardized methods, we vaccinated mice against GFP-HY using comparable amounts of LV-MHCII and of a vector pseudotyped with VSVg. Contrary to the broadly-interacting LV-VSVg, we observed a restricted and selective biodistribution of the LV-MHCII vector which essentially targeted DC in peripheral lymphoid organs, eliciting functional Th1 T cell responses and Tc1 effector immune response with establishment of memory. The MHC II-targeted LV may therefore represent a potentially safe alternative to limit off-target gene expression during gene-based vaccination.

## Materials and Methods

### Construction and plasmids

**Figure 1:**
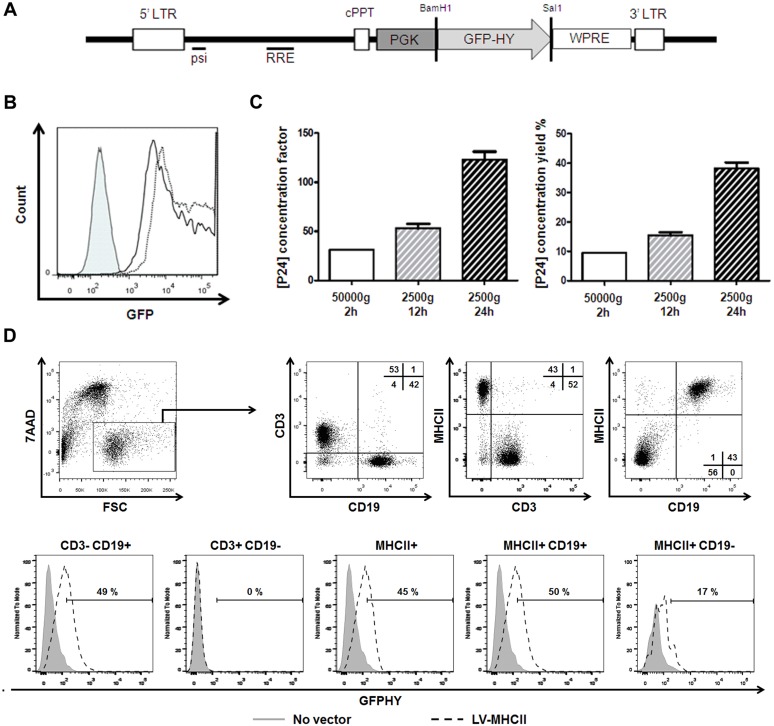
Characterization of the LV model. A. Schema of the transfer plasmid. The pRRL-PGK-GFP-HY transfer plasmid used for the production of LV-MHCII or LV-VSVg contains a heterologous RSV/HIV 5′ long-terminal repeat (LTR) and 3′ deleted LTR, psi encapsidation and Rev response element (RRE) sequences, a human phosphoglycerate kinase 1 gene (PGK) promoter, GFP-HY transgene, a woodchuck hepatitis virus post-transcriptional regulatory element (WPRE). BamH1 and Sal1 sites used for cloning are shown. B. Representative expression of GFP obtained from GFP-HY constructs. Result from 2 experiments showing expression of GFP by flow cytometry in 293T cells transfected with pRRL-PGK-GFP-HY (solid line, Mean Fluorescence intensity (MFI): 25683) or with the pRRL-PGK-GFP plasmid containing the native GFP (dotted line, MFI: 56779) or without plasmid. C. Production of LV-MHCII vector according to different concentration protocols: 50000 g 2 hours 4°C (n = 1), 2500 g 12 hours 4°C (n = 3) or 2500 g 24 hours 4°C centrifugation (n = 5) and measured as P24 concentration factor (left panel) or P24 yield (right panel) after P24 titers were measured on harvested stock and concentrated product. P24 concentration factor and yield were calculated for each condition tested n = 1 to 5. D. Transduction specificity *in vitro*. Freshly isolated C57Bl/6 mice splenocytes (5.10^5^ cells per well) pre-stimulated with IL2, IL4, IL7 overnight and the next day, LV-MHCII (50 ng RT/well) was added to the culture or not. Two days later, the expression of GFP-HY and MHC class II were measured by multi-color flow cytometry on the live population (7AAD- cells) and/or on the transduced cells in 3 separate experiments. Plots and histograms show additional CD3 and CD19 staining performed in 2 of these experiments.

The GFP-HY gene expression cassette coding for the enhanced green fluorescent protein (GFP) and T cell epitopes of the murine HY gene (Dby peptide (NAGFNSNRANSSRSS) presented by I-A^b^ and of the Uty peptide (WMHHNMDLI) presented by H2-D^b^) was obtained by multi-step fusion PCR (1) to create the annealing sites on GFP and HY sequences from respectively the pRRLsincPPT-PGK-GFP-WPRE plasmid (Primers: GFP F: 5′-ACCGAATCACCGACCTCTCC-3′ and GFP R: 5′-TTAAATCCAGCATTTGCAGAACCACTGCTCTTGTACAGCTCGTCCATGCCGAGAGTG-3′) and the pAAV-alphasarcoglycan-HY plasmid (primers: HY F: 5′-AGCAGTGGTTCTGCAAATGCTGGATTTAA-3′ and HY R: 5′-GCGTCGCAATTGGTCGACCTAGGTATTGTCTCCAATTAGATCCATATTATGGTGC-3′) [9], [10]; (2) to anneal and amplify the resulting PCR products with the GFP F and HY R primers and (3) to clone the resulting GFP-HY sequence into BamHI and SalI sites of pRRLsincPPT-PGK-GFP-WPRE plasmid thereby replacing the GFP sequence and generating the pRRLsincPPT-PGK-GFP-HY-WPRE plasmid represented in Figure 1A. Plasmids encoding measles glycoprotein envelope pFΔ30 and pHmutΔ18-MHC-II were used to produce the LV-MHCII vector and have been described previously [7], [8], [11]. All plasmids were produced and purified using the endotoxin-free Nucleobond PC2000EF kit from Macherey-Nagel (Düren, Germany).

### Lentiviral vector production and titration

Batches of recombinant HIV-1-derived lentiviral vector pseudotyped with MHCII-targeted MV glycoproteins (LV-MHCII) and encoding GFP-HY, were produced following transfection of HEK293 T cells with 5 plasmids using per 15 cm dish: 16 µg of each of the 2 envelope plasmids (pCG-FΔ30 and pCG-HmutΔ18-ScFV-MHCII); 5.6 µg of pKrev encoding the HIV-1 rev gene; 14.6 µg of pKLgagpol encoding the HIV-1 gag and pol genes and 16 µg of the transfer plasmid pRRLsincPPT-PGK-GFP-HY-WPRE. Medium was changed the day following transfection and collected 24 hours later to harvest the produced viral particles which were purified by centrifugation, as indicated in Figures. Particles were infectious for A20 murine B cell as reported [7] but infectious titration was not standardized and not systematically performed. Instead, all batches of LV-MHCII vectors were titered by physical titers measuring P24 and reverse transcriptase (RT) levels by ELISA (respectively Perkin Elmer, Waltham, MA, USA and Roche Applied Science, Indianapolis, IN, USA). Batches of recombinant HIV-1-derived LV pseudotyped with VSVg and encoding GFP-HY (LV-VSVg) were produced by transient transfection of HEK293T cells with 4 plasmids; 3 of which are described above (pKrev, pKLgagpol and pRRLsincPPT-PGK-GFP-HY-WPRE plasmids) and the fourth, pMDG encoding the vesicular virus glycoprotein G. LV-VSVg particles were purified by ion exchange chromatography capture as described [12] followed by concentration by ultracentrifugation (50 000 g for 2 hours). Infectious titers of LV-VSVg were determined as infectious genomes (IG) per ml using HCT116 cells as previously described [9]. Physical titers (P24 and RT) of LV-VSVg were also determined, showing 2.45×E+04 IG/ngP24 and 1.5×E+05 IG/ngRT for the batch used in this study. Virus-like particles (VLP) were produced similarly as above but omitting the glycoprotein envelope plasmid during HEK293T cell transfection. VLPs were purified and concentrated by a single step of ultracentrifugation (50000 g, 2 hours, 4°C). Physical titers of VLP were determined by P24 and RT ELISA.

### Mice

C57Bl/6 (CD45.2) and congenic CD45.1 (PtprcaPep3b/BoyJ [CD45.1]) mice were purchased from Charles River Laboratories (L’Arbresle, France), housed in our facilities under specific pathogen-free conditions and handled in accordance with French and European directives and under GMO L2 and A1 biological containment (agreement 5244-CAI).

### Ethics statement

Animal protocols were approved by the Genethon ethical committee and conducted by certified operators under agreement number CE12-037.

### Cells

Spleen cells for ELISPOT analysis were obtained by mechanical disruption of organs followed by red blood cell removal with ammonium chloride/potassium (ACK) lysis.

For flow cytometry, cells from thymus, spleen or lymph nodes were obtained following enzymatic digestion. Individual organs (thymus, spleen and lymph node) were diced and the resulting tissue fragments were cleaned by agitation in RPMI medium for 10 minutes at 4°C, then digested with a solution containing collagenase IV (1 mg/mL, Invitrogen) and Dnase I (50 µg/mL, Roche) for 15 minutes at 37°C under 900 rpm agitation. Cells released in suspension were collected and treated with 100 mM EDTA to stop the enzymatic reaction. The remaining non-digested tissue samples were incubated with a solution of freshly-prepared enzymes. The digestion procedure was repeated for a total of 3 times. Cells suspensions were washed in phosphate buffered saline (PBS) and an additional ACK lysis buffer step was added to remove residual red blood cells in thymic or splenic samples. Cell samples collected at the different steps of tissue processing were analyzed separately to measure GFP expression by flow cytometry whereas all cellular fractions obtained from the digestion of each organ were pooled together for flow cytometric cell sorting.

### Immunization

6 week-old mice were injected intravenously (IV) in the tail vein with vector diluted in PBS (from 10 to 100 ng of RT per mouse, as indicated) and control animals received equivalent volumes of PBS. For peptide challenge, a mixture of Dby-Uty peptides was administered intramuscularly at a dose of 100 µM (synthesized by Genepep, Montpellier, France) complexed with the same volume of incomplete Freund’s adjuvant (IFA) (Sigma Aldrich, Saint-Louis, MO). Controls were injected with PBS-IFA complexes.

### Genomic DNA extraction and real-time quantitative-PCR

Genomic DNA was extracted from frozen organs or from freshly sorted cells using the Wizard Genomic DNA Purification Kit (Promega, Madison, USA) following manufacturer’s instruction. Real-time PCR to amplify GFP-HY in relation to the titin gene was performed using ABI PRISM 7700 system (PE biosystems) with 0.2 mM of each primer and 0.1 mM of the probe according to the protocol Absolute QPCR Rox Mix (ABgene, Cambridge, UK). The primer pairs and Taqman probes used for GFP-HY and titin gene amplification were:

GFP-HY-F: 5′-ATGGTCCTGCTGGAGTTCGT-3′.

GFP-HY-R: 5′-TGCAGAACCACTGCTCTTGTACA-3′.

GFP-HY-P: 5′-ACCGCCGCCGGGATCACTC-3′.

mTitin-F: 5′-AAAACGAGCAGTGACGTGAGC-3′.

mTitin-R: 5′-TTCAGTCATGCTGCTAGCGC-3′.

mTitin-P: 5′-TGCACGGAAGCGTCTCGTCTCAGTC-3′.

Results were calculated from a standard curve based on dilutions of 2 plasmids containing either the GFP-HY or mouse TTN sequences. Background levels were comprised between 36–40 CT values.

### IFNγ ELISPOT

IFNγ ELISPOT assays were performed as previously described [13]. Results of spot forming units (SFU) for each mouse were calculated as the average value of duplicate measures after subtraction of background values obtained from the same cell sample in the absence of *in vitro* peptide stimulation.

### *In vivo* killing assay

Splenocytes from congenic male and female C57Bl/6 CD45.1 mice were incubated in obscurity at room temperature respectively with 0.2 µM or 2 µM of CFSE. After reaction stop and wash, cells were resuspended in PBS at specific concentrations (5×10^6^ CFSE-low male cells + 5×10^6^ CFSE-high female cells per 100 µL), then injected in the tail vein of C57Bl/6 CD45.2 mice (100 µL/mouse). Splenocytes from injected mice were collected 42 hours post-injection and the specific lysis of male target cells compared to female cells was analyzed by flow cytometry.

The % of male target cells lysis was assessed and calculated according to the following formula:

% of lysis = ([(average percentage of CD45.1 male cells/average percentage of CD45.1 female cells)_PBS_−(average percentage CD45.1 male cell/average percentage CD45.1 female cells %)_LV_)]/(average percentage of CD45.1 male cells/average percentage of CD45.1 female cells)_PBS_])*100.

### Flow cytometry

All reagents used for flow cytometry were purchased from BD-Biosciences (Le Pont de Claix, France). Cell suspensions were first incubated with anti-FcγRIII/II (2.4G2) monoclonal antibodies (mAbs), then stained in PBS with 0.1% bovine serum albumin (BSA) using saturating amounts of the following mAbs: phycoerythrin-conjuguated (PE) anti-CD3 (BD-Biosciences), biotinylated anti-CD19, biotinylated anti-CD11c, biotinylated anti-CD45.1 (eBioscience, San Diego, USA), PE-conjuguated anti-CD11b (BD-Biosciences), allophycocyanin-conjuguated MHC-II (eBioscience, San Diego, USA) and PE-cyanin 7 conjuguated streptavidin (BD-Biosciences). Dead cells were excluded using 7-actinomycine D (Sigma Chemical Co, St., MO) staining. Cells were analyzed on a LSRII cytometer (BD-Biosciences) using Flowjo or Diva software (BD-Biosciences) or sorted on Mo-Flo cell sorter (Beckman-Coulter). Sorted cells were collected in PBS 3% FBS, washed and suspended in PBS for genomic DNA extraction.

### Cytometric bead array

Splenocytes were added in duplicate wells in round bottom plates (1×10^6^ cells/well) and cultured in the presence of Dby or Uty peptide (2 µM) during 48 hours. Interleukin (IL) -2, IL-4, IL-6, IL-10, IL-12p70, IL-17A, TNFα, IFNγ and GM-CSF secretion by splenocytes was quantified by flow cytometry (LSRII) using the corresponding flex set of cytometric bead array kit (BD-Biosciences) using the manufacturer protocol and the FCAP software (BD-Biosciences).

### Statistical analysis

Significant differences between mean values were determined using unpaired two-tailed Student’s test upon verification of normal distribution of data using GraphPad Prism software. When indicated, one-way ANOVA followed by Bonferroni multiple comparison test was also used. P value under 0.05 was considered statistically significant.

## Results

### A novel model to follow transduced cells and anti-transgene immune responses

To evaluate gene-specific immunization in relation to the transduction of APCs, we constructed the GFP-HY transgene which is a fusion protein allowing simultaneous expression of a fluorescent marker (GFP) and of T cell epitopes Dby and Uty from the male HY gene product in transduced cells (Figure 1A). Fluorescence of GFP-HY was measurable by flow cytometry as shown by transfection of HEK293T cells (Figure 1B) although the signal intensity was slightly reduced compared to that of native GFP. Correct processing of T cell epitopes encoded by the construct were first verified by GFP-HY plasmid injections to mice in controlled immunization tests (data not shown) and detailed in subsequent experiments with viral vectors. The GFP-HY transgene model is therefore functional to track cells expressing and presenting the transgene, and to follow transgene-specific T cell immunization in C57Bl/6 mice.

### MHC-II targeting vector concentration optimization and validation

To comparatively immunize with vectors pseudotyped either with a ScFv specific for MHC class II (LV-MHCII) or with the broadly-interacting VSVg (LV-VSVg), it was necessary to improve the yield of LV-MHCII vectors from published techniques [7]. Vectors are produced by transient transfection and concentrated by centrifugation. Three different protocols varying in speed and time of centrifugation were compared. Protocols used for VSVg-pseudotyped LV concentration (50 000 g for 2 hours) gave very low yields of LV-MHCII vector, whereas the best results were obtained by centrifugation at 2500 g during 24 hours at 4°C (Figure 1C). This condition gave average titers of 5294±2782 ng P24/ml and 2341±1487 ng RT/mL (Table 1) thus increasing by 2 and 6 fold respectively the amount of P24 and RT recovered compared to the original protocol, which was sufficient for subsequent studies. The infectivity and specificity of the concentrated LV-MHCII vector was confirmed following transduction of murine A20 B cells (data not shown) and of spleen cells from C57Bl/6 mice *in vitro*. Spleen cells were incubated with the vector following a short pre-activation with IL2, IL4, IL7 to facilitate lymphocyte survival and transduction. Two days after LV-MHCII infection, GFP-HY expression was found only in MHC-II+ cells (representing 32±8% GFP+ cells in this cell population (n = 3)) which contain mainly CD19+CD3− B cells (Figure 1D). A small population of CD3−CD19− cells was also transduced, presumably corresponding to myeloid cells (macrophages and DC) which are known to be transduced by LV-MHCII in protocols without pre-stimulation [7]. These results confirm the preferential transduction of MHC-II+ cells by LV-MHCII and the preferential transduction of B cells within the MHCII+ cell population *in vitro,* as already established [7].

**Table 1:** LV-MHCII vector production titers.

		ng P24/mL	ng RT/mL
Harvested stock	n = 9	49±29	9±5
Concentrated LV	2500 g 12 h 4°C n = 3	3539±2819	397±95
	2500 g 24 h 4°C n = 5	5294±2782	2341±1487

Virus particles were produced by transient transfection of 293T cells and the harvested stock was concentrated using the indicated conditions of centrifugation. The concentrated LV was 0.45 µm-sterile filtered, cryopreserved (−80°C) and subsequently titered for P24 or RT content by ELISA. n = number of independent productions.

### LV-MHCII induce a lower, but functional, Th1- polarized immune responses than LV-VSVg

**Figure 2:**
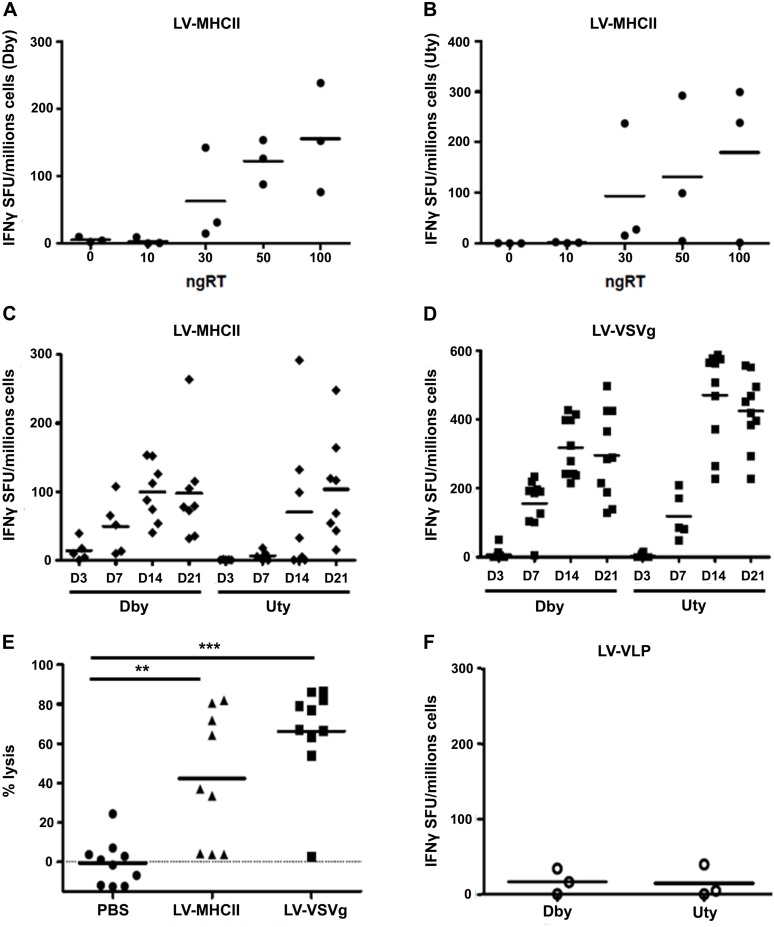
Immune responses induced by IV administration of LV with different pseudotypes. C57Bl/6 female mice were injected IV in the tail vein with PBS, LV-VLP, LV-VSV or LV-MHCII vectors. Immune responses were measured by IFNγ ELISPOT following a 24-hour-Dby or Uty ex vivo stimulation of splenocytes (**A** to **D** and **F**) or a *in vivo* cytotoxic assay (**E**). A. and B. Dose response curves with LV-MHCII. Each mouse received IV 0, 10, 30, 50 or 100 ng RT and 14 days later, spleen cells were collected and IFNγ ELISPOT was performed following Dby (a) or Uty (b) stimulation. Each symbol represents IFN-γ spot-forming unit (duplicate measures) of each mouse (n = 3 mice per group). C. and D. Kinetics of response to 50 ng RT of vector. Immune response to LV-MHCII (C) or LV-VSVg-GFP-HY (D) was measured by IFNγ ELISPOT 3, 7, 14 and 21 days after injection and following Dby or Uty peptide stimulation as indicated (n = 2 separate experiments, 5 to 10 mice per group). E. *In vivo* cytotoxic assay. C57Bl/6 mice were injected with 50 ng RT of LV-VSVg or LV-MHCII. 13 days later, a mix of congenic C57Bl/6 CD45.1 male CFSE^low^-labeled cells and female CFSE^high^-labeled cells was injected to the previously immunized mice (10^7^ cells per mice) to measure the specific killing of male cells by flow cytometry (n = 2, 10 mice per group). Statistical analyses using Student’s t Test or ANOVA with Bonferroni multiple group comparison show significance indicated with ** for p values<0.01 and *** for p values<0.001. F. Lack of immunization from VLP. 50 ng RT of LV-VLP were injected per mice and 14 days later immune responses were measured by IFN-γ ELISPOT (n = 3 mice per group).

**Figure 3:**
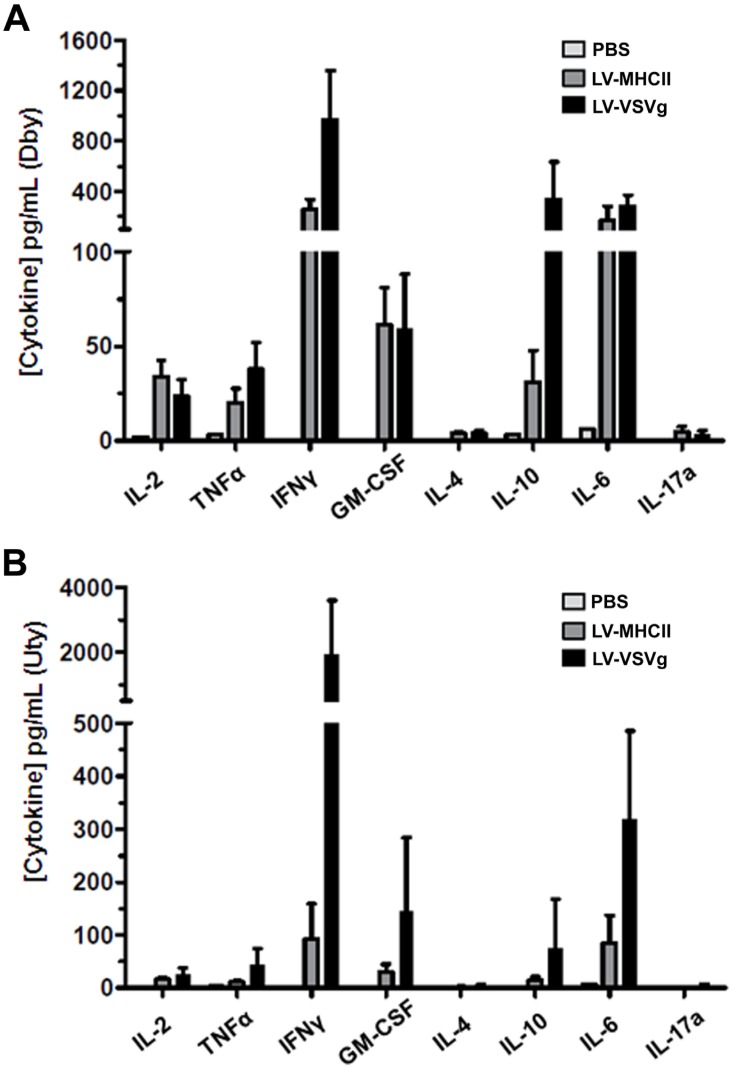
Cytokines secreted following immunization. C57Bl/6 female mice were injected IV with PBS, 50 ng RT of LV-VSVg or LV-MHCII vectors. 7 days later, spleen cells of these mice were harvested and assayed for IL-2, IL-4, IL-6, IL-10, IL-17A, IFNγ, TNFα and GM-CSF secretion after a 48 hour stimulation ex vivo with Dby (**A**) or Uty (**B**) using the cytokine bead array (n = 5 mice per group). Data and assay limits of detection are displayed in Table 2.

Dose-response curves in mice showed that a single intravenous (IV) injection of at least 30 ng RT of the LV-MHCII vector reproducibly induced HY-specific CD4+ and CD8+ T cell IFN-γ production from spleen cells in ELISPOT assays (Figure 2A and 2B), thus confirming the immunizing dose range of the vector established with the ovalbumin transgene model [7]. All subsequent experiments with GFP-HY to further characterize T cell immunization used 50 ng RT of vector per IV injection. Dby-specific CD4+ T cell responses appeared 7 days post-injection (Figure 2C) and Uty-specific CD8+ T cell anti-transgene immune responses (Uty) were clearly seen after 14 days (Figure 2D). The same amount of LV-VSVg vector (50 ng RT/mouse IV) induced comparable kinetics of IFNγ-producing CD4 and CD8 T cell responses but reached respectively 3 and 10 fold higher IFN-γ levels than with LV-MHCII injections (Figures 2C and 2D). In spite of a low CD8 IFNγ T cell response, the LV-MHCII vector triggered functional cytotoxic immune reactions leading to specific and significant killing of male target cells injected into mice (Figure 2E). The levels of cytotoxicity were comparable, although slightly reduced compared to LV-VSVg but this did not reach statistical significance. Immunization by GFP-HY vectors was specific and directly caused by the entry of particles into cells in mice and not by passive transfer of GFP-HY protein contaminants in vector preparations. Indeed, the injection of 50 ng RT of virus-like-particles (VLP) produced without envelope pseudotype, but otherwise generated and concentrated similarly as fully-infectious particles, did not trigger CD4 or CD8 T cell responses (Figure 2F). Using cytometric bead arrays following a short *ex vivo* re-stimulation of spleen cells by Dby or Uty peptides, we found comparably high levels of CD4 T cell cytokines IL-2, GM-CSF and IL-6 after LV-MHCII or LV-VSVg injection (Figure 3A, Table 2). Cytokines IFNγ, TNFα and IL-10 were also induced but at lower levels with LV-MHCII. None of the vectors triggered IL-4 or IL-17 production in conditions used. For CD8 T cell cytokines, it was clear that LV-VSVg induced higher levels of IFN-γ, GM-CSF and IL-6 than LV-MHCII but the induction of IFNγ in CD8+ T cell by this vector was (Figure 3bB, Table 2) consistent with ELISPOT results (Figures 2B and 2C).

**Table 2:** Cytokine quantification by cytometric bead array technology following *ex vivo* spleen cell restimulation with peptides.

		IL-2	IL-4	IL-6	IL-10	IL-12p70	IL-17a	TNFα	IFNγ	GM-CSF
Dby restimulation	PBS	2,2±1,2	0,7±0,6	6,1±3,8	<9,6	<1,9	<0,95	3,4±1,8	<0,5±0,9	<1,5
	LV-VSVg	23,6±8,9	4,2±1,3	281,0±89,0	336,0±298,0	<1,9	2,5±3,0	37,9±14,3	967,0±393,0	58,6±29,6
	LV-MHCII	34,1±18,5	3,9±1,2	175,0±108,0	31,1±16,7	<1,9	4,6±3,0	20,0±7,6	256,0±81,0	61,5±19,5
Uty restimulation	PBS	2,3±1,7	<0,3±0,4	6,8±7,0	<9,6	<1,9	<0,95	3,1±0,7	<0,5±0,8	<1,5
	LV-VSVg	24,2±13,4	5,2±1,0	317,0±168,0	74,5±93,8	<1,9	3,6±3,8	44,5±29,3	1925,0±1669,0	144,0±141,0
	LV-MHCII	16,4±2,1	2,4±0,5	84,7±52,2	13,8±7,5	<1,9	1,1±0,8	11,0±2,6	92,6±66,6	29,8±15,1

The data presented here are illustrated in Figure 3 and represent averaged values of cytokine levels measured by CBA in 5 mice ± standard deviation. The limits of detections for each cytokine are: IL-2: 0.2 pg/mL; IL-4: 0.3 pg/mL; IL-6: 1.4 pg/mL; IL-10: 9.6 pg/mL; IL-12p70: 1.9 pg/mL; IL-17A: 0.95 pg/mL; IFNγ: 0.5 pg/mL; TNFα: 2.8 pg/mL and GM-CSF: 1.5 pg/mL.

### LV-MHCII elicits T cell memory against the transgene

**Figure 4:**
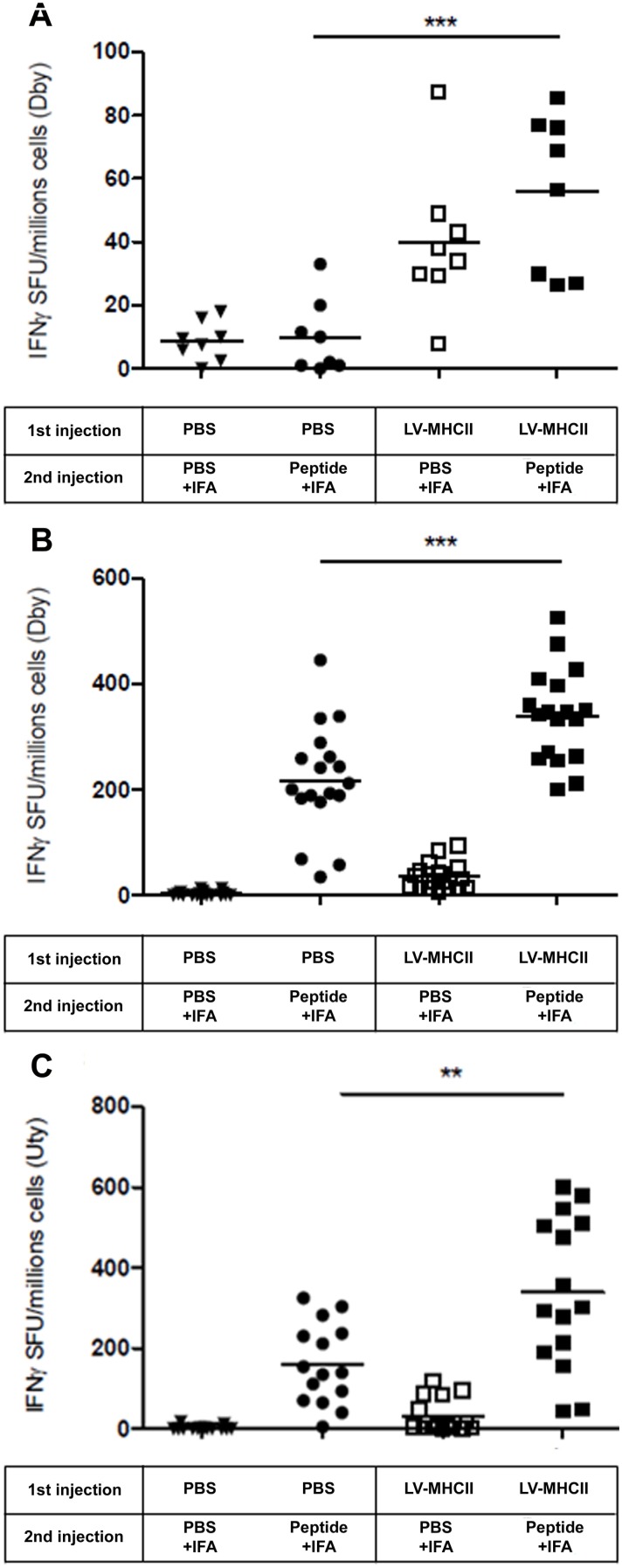
Induction of a transgene-specific memory response. C57Bl/6 female mice were injected IV with PBS or LV-MHCII. 35 days later, mice were challenged by an intramuscular injection of a mixture of Dby and Uty peptides complexed with IFA or as control with PBS complexed with IFA. IFNγ secretion of splenocyte was assayed **A**. 5 days after the challenge following a 24 hour Dby restimulation. **B**. 14 days after the challenge following a 24 hour Dby restimulation or **C**. Uty restimulation. Each symbol represents IFN-γ spot-forming unit (duplicate measures) of each mouse from 2 independent experiments (n = 8 mice per group). Statistical analysis shows ** for p values<0.01 and *** for p values<0.001.

The establishment of T cell immune memory was assessed 40 to 49 days after a single injection of vector. Memory CD4 and CD8 T cell responses were measured by IFNγ ELISPOT following a challenge at day 35 with the Dby and Uty peptides complexed with incomplete Freund’s Adjuvant (IFA). Control mice were challenged with PBS complexed with IFA. The vector augmented both the early and late memory T cell responses. The early memory response occurred only 5 days post-challenge at a time when primary responses to peptides with IFA were not detected (Figure 4A). The late CD4 and CD8 T cell memory response was evident 14 days post-challenge (Figure 4B, 4C). At such time, mice injected with vector but challenged with PBS did not respond confirming that this was indeed a read-out of T cell memory and not the residual immune response from the initial vector injection (Figure 4B and 4C).

### The MHC-II targeting vector transduces DC in lymph nodes *in vivo*

**Figure 5:**
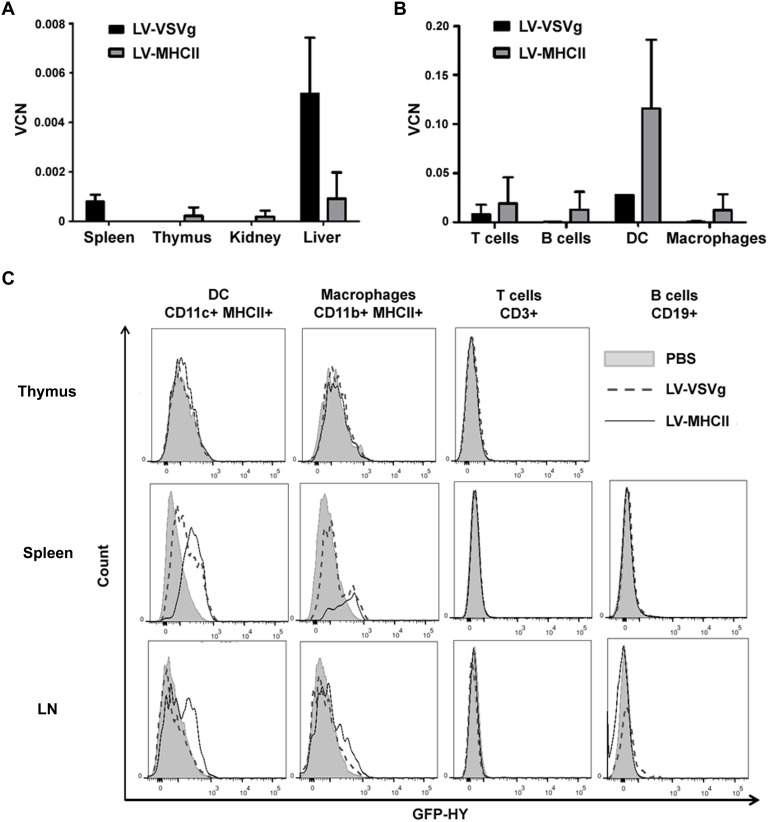
Evaluation of vector biodistribution and the vector-targeted cell population. C57Bl/6 female mice were injected intravenously with PBS, 50 ng RT of LV-VSVg or LV-MHCII. 3 days later, spleen, thymus, lymph nodes, kidney and liver from these mice were harvested. **A**. The vector copy number (VCN) per cell was assessed by qPCR on genomic DNA extracted from spleen, thymus, kidney, liver (n = 5 mice) and **B**. from sorted spleen population with GFP-HY specific Taqman probes (n = 2) **C**. GFP-HY expression in the thymic, splenic and lymph node immune cells was assessed by flow cytometry. Thymic, splenic and lymph node cells were marked by CD3, CD19, MHC-II, CD11b or CD11c to identify macrophages, DC, T and B cells. GFP-HY fluorescence for each population is shown (n = 2).

Immunization was correlated with the biodistribution of vector and transgene expression *in vivo*, by measuring the presence of vector and transgene expression in various organs and cells of mice using qPCR and flow cytometry, 3 days following IV injection of 50 ng RT of vector per mouse. This time point was based on published studies with Sindbis virus-pseudotyped LV detecting transgene-expressing DC in lymph nodes after 3 days [4]. Later time points (day 7, 14, 21) revealed less vector signal than at day 3 (not shown). In these conditions, injecting LV-MHCII led to undetectable vector signal by qPCR in liver, spleen, thymus and kidney organs of mice (Figure 5A). However, a signal became apparent following subcellular fractionation of spleens using enzymatic digestion and cell sorting., LV-MHCII vector copies were found in the purified CD11c+ CD11b− DC population of splenocytes whereas the qPCR signal was barely detectable in purified CD3+ CD19− T cells, CD19+ CD3− B cells and CD11b+CD11c− macrophages (Figure 5B). Transgene detection was confirmed by FACS in CD11c+ MHC II+ DC and to a lower level in CD11b+ macrophages of lymph nodes and spleen but not in T or B cells of any organ tested (Figures 5B and 5C). The very low level of transgene expression found in CD11b+ macrophages while copies of vector were not detectable in these cells suggests possible passive pick-up of transgenic proteins. In contrast with such restricted detection of LV-MHCII vector, a distinct and broad biodistribution of the LV-VSVg vector was evident since copies of vector were found essentially in the liver with a low signal in the spleen. Spleen cellular fractionation showed only a small signal in CD11c+ DC by qPCR (Figure 5B) confirmed by transgene expression but not clearly in other cell populations (Figure 5C). The difference between the vector signal found in total spleen and the low signal in spleen cell subsets suggests that the LV-VSVg may have tranduced parenchymal tissue cells, consistent with prior reports [14], [15]. Overall, in conditions used, and in contrast with more broadly-distributed vectors, the LV-MHCII targets preferentially the small population of CD11c+ DC in peripheral lymphoid organs, but not other cell populations including B cells.

## Discussion

Targeted cell entry is a desirable goal in many gene transfer applications [6], [11], [14] as well as in vaccination strategies [3]. Our data show that vectors targeting MHC class II for entry could represent a novel strategy to preferentially transduce DC *in vivo*, resulting in robust transgene-specific T cell immunization with limited vector biodistribution and circumscribed transgene expression. While LV-VSVg have already been used as vaccines [2], relatively few strategies have been reported to direct LV to APCs but there is a growing interest for such specific approaches. Redirection of LV tropism to DC by pseudotyping was first reported with the use of Sindbis virus glycoproteins recognizing DC-SIGN to target DC [4]. More recently, strategies using fusion-defective VSVg complexed to DC-specific camel Ig sequences called nanobodies were also used to target subpopulations of murine DC or macrophages as well as human DC [5], [16], [17]. Nanobody-targeted LV recognizing various types of DC, and more specifically CD11c+ conventional DC, induce effective T cell immunization following intranodal administration in mice [17]. However, intranodal administration is not a stringent model as it allows the induction of T cell responses even with the use of non infectious LV. Using a systemic route of administration via the tail vein to deliver LV with engineered MV glycoproteins, we herein show that it is possible to target and to transduce CD11c+ DC to immunize the mice. In this stringent system, the possibility of indirect immunization due to LV production contaminants is excluded by the lack of effects of entry-defective VLPs. Thus, by extending the initial description of the LV-MHCII vector [8] [7] with in vivo bio-distribution studies done with purified particles and with the use of a novel antigenic model, our study provides a first demonstration that it is possible to raise functional antigen-specific CD4 and CD8 T cell responses via preferential gene transfer in CD11c+ DC.

The *in vivo* biodistribution and cell tropism of the LV-MHCII was selective. Following systemic administration, the vector was found preferentially in lymphoid organs and more specifically in CD11c+ DC while T or B cells were not targeted. The lack of B cell transduction *in vivo* was contrary to expectations from *in vitro* experiments in the present study (Figure 1D) and in prior published reports [7], [8]. Even if they express MHCII, the majority of B cells are in a resting state *in vivo* and may be less permissive to LV transduction than when cultured. Entry into cell cycle is not necessarily required for B cell transduction by LV but this effect is pseudotype-dependent [18]. LV-VSVg also failed to transduced B cells *in vivo* perhaps due to their resting state as well. Low density lipoprotein receptors recently identified as VSVg receptors [19] are scarce on resting human B cells but levels are augmented following activation thereby allowing efficient B cell transduction by VSVg-pseudotyped LV [20]. Additional factors may also interfere with *in vivo* vector entry in B cells *in vivo*. Alternatively, B cell transduction *in vivo* may be limited by low bioavailability of vector in lymphoid organs. Upon intravenous injection, LV-VSVg will go primarily to the liver (more precisely into Kupffer cells) and also in bone and spleen [14], while the targeted vector probably remains in the circulation and can reach lymph nodes. This could explain why LV-VSVg essentially transduced DC and macrophages in the spleen but not in lymphoid organs whereas LV-MHCII was able to transduced DC and some macrophages in spleen but also in lymph nodes.

The preferential targeting of DC by LV-MHCII led to a robust T cell response and this was achieved with relatively modest amount of vector. A single non-adjuvanted intravenous injection of 50 ng of RT of LV-MHCII vector per mouse was sufficient to establish effector CD4 and CD8 T cell responses and durable T cell memory. The use of viral vectors for immunization provides not only the transgenic antigen to APCs but may also deliver some activating signal to the APC. This was shown with Sindbis Virus modified envelope which not only target cells expressing DC-SIGN but also provide secondary signals to DC via ligand recognition that facilitate immunization [4] [21]. LV with engineered MV glycoproteins directed to CD19 or to CD20 are known to provide a low level of activation to target cells which facilitates their transduction [18]. It is therefore possible that LV-MHCII provide a secondary activation signal to transduced cells facilitating the generation of functional cytolytic T cell responses and T cell memory which require cross-presentation and T cell help. The nature of this second signal remains unclear. A role of contaminants can be minimized in our system because vectors were purified and produced in endotoxin-free conditions. Mechanisms of LV entry and of reverse transcription in DC may contribute to this activation as shown previously [22].

While LV-MHCII can be used as efficient vaccines, the results are surpassed by those of LV-VSVg which induce much higher levels of CD4 and CD8 T cell responses in mice. This superiority was already reported in another system comparing LV-VSVg with DC nanobody-targeted LV [17]. However, the efficacy of LV-VSVg comes at the cost of a broad insertion of the transgene in the organism including high levels in the liver as expected from the broad tropism of the VSVg pseudotype [14], [15]. By transducing not only hematopoietic cells but also stromal tissue, LV-VSVg probably allow a broad antigenic presentation of the transgene on MHC class I which contributes to the high CD8+T cell responses observed with this pseudotype. However, if such broad transgene expression may amplify immune reactions, it also creates tissular targets susceptible to cytolytic destruction, inflammation or auto-immunity as described in other systems [23]. Transduced solid tissues may also induce immune modulation as suggested by the production of IL-10 observed following LV-VSVg immunization (Figure 3A). Thus, by inducing high Th1/low IL-10 cytokine responses, LV-MHCII may effectively promote cytolytic T cell development in spite of a relatively restricted MHC class I antigenic presentation. One major advantage of LV-MHCII vectors is that they reduce off-target transduction which is a gain in terms of specific activity and a real safety advantage. Administering the LV-MHCII vector to mice results in very little genomic off-target integration in the body, as tracked by qPCR, thereby reducing the risk of LV genotoxicity linked to insertional mutagenesis [24]. This risk is further reduced by the short life-span of the target cells as DC or macrophages are mature end-stage cells. However rare integration events in non-target cells cannot be formally excluded. Non-integrative LV could be proposed as an alternative to improve the safety of genetic vaccine strategies but much larger doses may be required to achieve sufficient immunization [1].

Our data support the notion that DC-targeted gene transfer allows specific vaccination with reduced off-target effects and at low antigenic doses. Based on these results, further development and testing of envelope-engineered LV seems warranted in the vaccination field. These tools provide additional solutions for DC-targeted vaccines besides targeted liposomes or antibody-coated nanoparticles [3]. With this example of DC-targeted vaccines, our results also diversify the possible applications of the MV glycoprotein LV pseudotype platform [14], [25].
